# CONTINGENCY PLANNING AND STYLES OF PREPARATION FOR FUTURE CARE BY OLDER GRANDPARENT CAREGIVERS

**DOI:** 10.1093/geroni/igad104.3099

**Published:** 2023-12-21

**Authors:** Tina Peterson

**Affiliations:** University of Cincinnati, Cincinnati, Ohio, United States

## Abstract

The COVID-19 pandemic heightened attention to contingency planning in every area of life including family caregiving. Extended time in the caregiver role can increase problem-solving abilities; yet low rates of traditional contingency planning exist among custodial grandparents. This research applied Steele and colleagues’ four styles of preparation for future care (2003)—avoider, thinker, planner, or consenter—to older custodial grandparents raising adolescent grandchildren. Nineteen grandparents participated in face-to-face interviews. Criteria were: primary caregiver for a grandchild 12 or older; grandchild in home at least 3 days weekly; and grandparent 40 or older. Recruitment included word of mouth, newspapers, and flyers. Participants were asked “What is your thinking about caregiving arrangements for your grandchild in case you are no longer able to provide care? What actual steps, if any, have you taken to begin these caregiving arrangements?” Interviews were audiotaped, transcribed verbatim, and analyzed using conventional content analysis. The racial and ethnic composition of the grandparents were white (n=10), Black/African American (n=7), Native American (n=1), and Filipino (n=1). Most respondents were married (58%), white (53%), grandmothers (84%), with some college education or higher (79%), and an average age of 66 years. Grandparents identified as thinkers (42%), planners (32%), consenters (21%), and avoiders (5%). Two married grandfathers reported conflicting preparation styles (thinkers) in comparison to their spouses (planners). Grandparents often relied on oral opposed to formal written plans. Findings suggest research, practice, and education is needed to assess factors inhibiting aspects of contingency planning for future care among thinkers and avoiders, particularly older grandfathers.

